# High specific surface area γ-Al_2_O_3_ nanoparticles synthesized by facile and low-cost co-precipitation method

**DOI:** 10.1038/s41598-023-33266-0

**Published:** 2023-04-15

**Authors:** Zahra Gholizadeh, Maryam Aliannezhadi, Mehrdad Ghominejad, Fatemeh Shariatmadar Tehrani

**Affiliations:** Faculty of Physics, Semnan University, P.O. Box: 35195-363, Semnan, Iran

**Keywords:** Nanoscience and technology, Physics, Applied physics

## Abstract

Alumina (Al_2_O_3_) nanoparticles (NPs) are particularly adsorbent NPs with a high specific surface area (SSA) that may well be utilized to clean water. In this study, pure γ-alumina NPs are successfully synthesized by the co-precipitation method, and the effect of ammonium bicarbonate concentration on the synthesized NPs is studied to find the optimum concentration to provide the highest capacity of copper ions removal from water. The results declare that spherical alumina NPs with average diameters in the range of 19–23 nm are formed with different concentrations of precipitation agent, and the concentration has no significant effect on the morphology of NPs. Furthermore, the precipitating agent concentration influences the optical characteristics of the produced alumina NPs, and the bandgap energies of the samples vary between 4.24 and 5.05 eV. The most important impact of precipitating agent concentrations reflects in their SSA and capacity for copper ion removal Ultra-high SSA = 317 m^2^/g, and the highest copper removal at the adsorbate concentration of 184 mg/L is achieved in an alkalis solution followed by a neutral solution. However, admirable copper removal of 98.2% is even achieved in acidic solutions with 0.9 g/L of the alumina NPs synthesized at a given concentration of ammonium bicarbonate, so this sample can be a good candidate for Cu ions removal from acidic wastewater.

## Introduction

The production of nanostructured metal oxide materials has recently received much attention due to their unique features, such as high surface-to-volume ratio, high surface reactivity, and unusual electric properties. Metal oxides are extremely used in electronic and photonic devices, medicine, and also as catalysts and photocatalysts. They found particular desirability in many scientific fields, including chemistry, material sciences, physics, medicine, and electronics^1–3^.

Aluminium oxide, commonly known as alumina, is one of the most frequent metal oxides used in industry. Alumina nanoparticles have wide usage in adsorption-based applications because of their interesting properties such as acid–base properties, high specific surface area (SSA), structural stability, low cost, mechanical and thermal stability, good mechanical strength, volatile acidity, thermal conductivity, stiffness, inertness to most acids and alkalis, adsorption capacity, wear resistance, oxidation, good electrical and chemical resistance, electrical insulation, high melting points, as well as being non-toxic. Among these features, the high surface area, and open porosity enable γ-alumina to be applied as catalysts and adsorbents in petroleum refining and petrochemical industries^4–8^.

Unlike organic pollutants, heavy metals are not decomposed naturally and incline to gather in living organisms and many heavy metal ions are the most common toxic contaminants mostly summarized in industrial effluents. Toxic heavy metals of particular attention in the treatment of industrial effluents include copper, nickel, lead, mercury, zinc, chromium, and cadmium^9^.

Copper ion (Cu^2+^) is one of the harmful heavy metals, which abundantly and naturally present in municipal wastewaters and industrial effluents, and is very harmful to human health^10^. Copper ions, as a toxic contaminant of potable water resources, should be eliminated because of their dangerous risks at unauthorized dosages (more than 2 mg/L) for human health problems such as headaches, depression, and learning problems^11^. Adsorption has been accepted as one of the most promising methods for the removal of toxic metals from aqueous solutions due to its simplicity, flexibility, and high efficiency in industrial applications^12^. The use of nanoscale materials in the field has attracted considerable attention due to their large specific surface area and excessive active groups.

Alumina nanoparticles (Al_2_O_3_ NPs) are important inorganic materials with a good adsorption capacity, high resistance to chemical agents, and excellent performance, which introduce them as a good catalyst candidate for many chemical reactions and the best candidate for water treatment. So, different research groups have synthesized alumina nanostructures and used them to remove a variety of contaminant ions from water. For example, S.M Siahpoosh et al. have synthesized alumina nanoparticles using the sol–gel method and exploited them to remove nickel contaminants from water^13^. Also, Mahdavi et al.^14^, Amin et al.^15^, and Shojaei Bahabad et al.^16^ have established nano alumina to remove some heavy metals such as Pb(II), Cu(II), Cd(II), and Ni(II) from aqueous solutions. Besides, Huimin Zhang et al.^17^ and Sara Al-Salihi et al.^18^ have used nano alumina to remove Congo red dye from water. Therefore, nano alumina is accepted as an excellent nanostructure for water treatment.

Alumina can be synthesized in several metastable transition phases depending on the preparation method as well as synthesis parameters like stirring times, calcination temperature, precursor, solution pH, the starting material, and also additive materials like organic additive and chelating agents^4,19^. A well-known group of alumina like η, χ, ρ, and γ-Al_2_O_3_, which labeled as Al_2_O_3_.n H_2_O (n is a number between 0 and 6), is created at low-temperature by dehydration of boehmite and bayerite at ~ 600 °C. Meanwhile, other alumina groups like θ, δ, κ, and α-Al_2_O_3_, which are labeled as anhydrous Al_2_O_3_, are mostly produced at high-temperature calcination in the range of ~ 900 to 1000 °C. Phase transition in alumina follows the sequence Boehmite → γ-Al_2_O_3_ → δ-Al_2_O_3_ → θ-Al_2_O_3_ → α-Al_2_O_3_. γ-alumina and α-alumina are the only types of alumina that are commercially produced until now^20,21^. Also, nano alumina can be synthesized by various routes, such as hydrothermal^22^, mechanical milling^23^, co-precipitation^24,25^, vapor phase reaction^26^, arc plasma^27^, sol–gel^21^, and homogeneous precipitation^28^. The co-precipitation method (CPM) is an easy, simple, and cost-effective synthesis method, which provides the ability to control the particle crystalline size, morphology, and numerous prospects of the NPs to customize the particle surface and properties by determining the relative rates of nucleation and growth during the synthesis process, especially when removing the solvent^29–31^. Also, different precipitating agents like sodium carbonate, sodium bicarbonate, ammonium carbonate, ammonium bicarbonate^32^, ammonium hydroxide^31^, and hexamethylene tetramine^33^, and different synthesis parameters have been used to synthesize high specific surface area (SSA) Al_2_O_3_ nano-powders. Some reported SSA and synthesis conditions of the nano alumina are collected in Table 1 for comparison.

**Table 1:** Method, precursor, precipitating agent, calcination temperature and time, and SSA of synthesized γ -Al_2_O_3_ nanoparticles.

Method	Precipitating agent	Precursor	calcination temperature and time	SSA (m^2^/g)	References
CPM	Sodium carbonate	Aluminum nitrate	550° (5 h)	140	^24^
CPM	Sodium bicarbonate	Aluminum nitrate	550° (5 h)	185	^24^
CPM	Ammonium carbonate	Aluminum nitrate	550° (5 h)	179	^24^
CPM	Ammonium bicarbonate	Aluminum nitrate	550° (5 h)	190	^24^
Green method	Ammonium hydroxide	Aluminum nitrate	650° (2 h)	217.02	^32^
Hydrothermal	Hexamethylene tetramine	Aluminum sulfate	650° (2 h)	273.3	^33^

As you can observe, the precipitating agents and synthesis route have a significant effect on the SSA of the synthesized nano alumina, so using NH_4_HCO_3_ in the co-precipitation method led to a higher value of nano alumina SSA than other precipitating agents. Therefore, this precipitating agent can be a good candidate to achieve high SSA nano alumina. Furthermore, the co-precipitation method is a simple, rapid, lower temperature, and low-cost method with the ability to easily control the sample crystalline size and morphology, well controllability over the stoichiometric, and overall homogeneity for synthesizing the nanoparticles.

So, the co-precipitation method is selected to synthesize the high SSA alumina NPs. Also, NH_4_HCO_3_ is selected as the precipitating agent because the highest SSA has been achieved by using this material. To the best of our knowledge, there is no evidence to investigate the effect of NH_4_HCO_3_ molar concentration on the properties of synthesized alumina nanoparticles or any effort to achieve the optimal alumina nanoparticles with the highest SSA for water treatment in the literature. So, the synthesis of Al_2_O_3_ nanoparticles using different amounts of NH_4_HCO_3_ is done in the paper by co-precipitation method to consider the effect of NH_4_HCO_3_ molar concentration on the characteristic properties of nano alumina and the removal of copper ions as a toxic metal from aqueous solutions. The results declare that the molar concentration of NH_4_HCO_3_ is a significant parameter that affects the properties of alumina NPs and Cu^2+^ ion removal from water.

## Material and method

### Synthesis

Aluminium nitrate (Al(NO_3_)_3_, 95%), Ammonium bicarbonate (NH_4_HCO_3_, 98%), sodium chloride (NaCl, 99%), and NaOH were purchased from Merck company and used without further purification.

For the synthesis of Al_2_O_3_ nanoparticles, 2.5 g of aluminium nitrate (Al(NO_3_)_3_) and a given mass of ammonium bicarbonate (0.6, 0.8, 1, and 1.2 g of NH_4_HCO_3_) were dissolved in 60 mL of deionized water (DI water) separately on two different magnetic stirrers at ambient temperature for 15 min. Then, two previous solutions were inserted into 40 mL of deionized water and taken in a reaction vessel on an adjusted magnetic stirrer at a temperature of 70 °C by two droppers to finish the solutions.

After that, 8 g of NaOH was dissolved in 100 mL of DI water for 15 min on a magnetic stirrer at ambient temperature and inserted into the previous solution with a dropper to adjust the solution pH to 8. The precipitate was left for 3 h on a magnetic stirrer adjusted at 70 °C. Next, Al cations precipitate in the form of hydroxides. The white precipitate was washed with DI water (three times) and ethanol (two times) to remove all impurities including Na ions and else. Then the alumina product was transferred to an oven at 70 °C for 12 h to dry.

**Figure 1:**
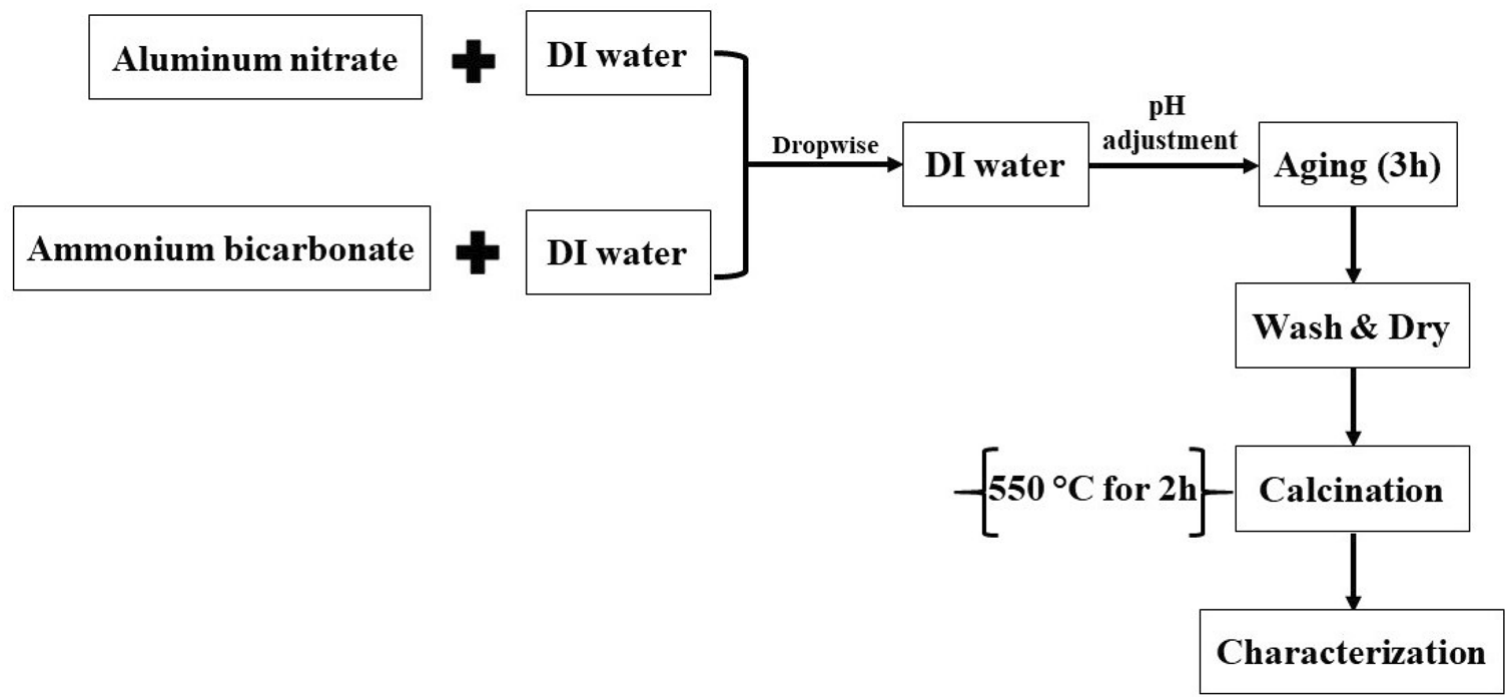
Flowchart of synthesis processes of alumina NPs.

Calcination of all alumina samples was carried out in a programmable furnace at 550 °C for 2 h with a temperature rate of 30 °C/min. The white calcined samples prepared using different masses of NH_4_HCO_3_ (0.6, 0.8, 1, and 1.2 g) were labeled according to the used mass of NH_4_HCO_3_ as RA-0.6, RA-0.8, RA-1, and RA-1.2, respectively. The flowchart of the synthesis process of alumina NPs is shown in Fig. 1.

### Characterization

Fourier transform infrared (FTIR) spectroscopy, Shimadzu spectrometer, model 8400S, was done by KBr disc technique in the range of 400–4000 cm^−1^ to investigate the structure, vibration modes, and the chemical bonding configuration of the alumina samples. The white powders were investigated by X-ray diffraction (XRD), ADVANCE-D8 model, with Cu-kα radiation source (λ = 1.5406 Å) in the range of 2θ = 10–90° to identify the phases and the crystallinity of the alumina calcined samples. Field emission scanning electron microscopy (FESEM) and dispersive X-ray analysis (EDX), TESCAN-model MIRA3 microscope, were used to study the surface morphology and elemental analysis of the samples. The optical properties of the alumina samples were investigated using diffuse reflectance spectroscopy (DRS) by the Avaspec-2048-TEC device. The specific surface areas (SSA) of the samples were estimated from Brunauer–Emmett–Teller (BET) theory by using nitrogen adsorption–desorption isotherm data obtained at − 196 °C (77 K) on a constant-volume adsorption apparatus with Micromeritics Gemini VII version 5.03. The samples were degassed at 200 °C for 3 h before BET analysis.

### Application

Adsorption of copper ions by synthesized nano-alumina was studied by taking out 55 mL of Cu^2+^ ion solution (184 ppm) at room temperature. For investigation of copper ion removal from water, 0.050 g of synthesized nano-alumina were dissolved in this acidic solution (pH = 5.8) on a magnetic stirrer and the adsorption reactions were studied using atomic absorption spectroscopy (AAS) on the solution after filtering and separation of the alumina NPs using a No. 4 sinter glass filter to evaluate the amount of copper in the water. Agilest Technologies 240AA flame atomic absorption spectrometer was used to obtain the AAS of the simulated water and determine the copper ions concentration in the solution. The nano-alumina sample with the best removal efficiency of Cu^2+^ in acidic conditions was further studied under the same experimental conditions in neutral and alkaline solutions (pH = 7 and 8), too.

To estimate the point of zero charge (PZC) for the synthesized alumina NPs, 0.05 g of each sample was added separately into 50 mL of 0.01 M NaCl solution (as background electrolyte) with various pH values from 2 to 10 and left for 48 h to reach the equilibrium. Then, the pH of the solution was measured using a pH meter and recorded to calculate the difference between the final and initial pH value of the solution which can be used to determine the PZC of every synthesized alumina NPs.

## Result and discussion

### FTIR analysis

**Figure 2:**
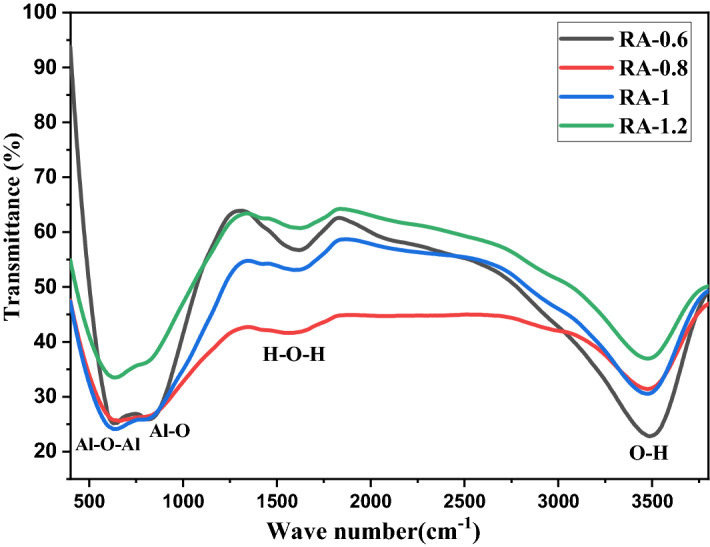
FTIR transmission spectra of the samples synthesized with different concentrations of ammonium bicarbonate including 0.6, 0.8, 1, and 1.2 g in 60 mL of deionized water.

Fourier transform infrared (FTIR) spectroscopy was performed on the alumina samples to investigate the vibrational states and chemical structure of the samples. Figure 2 shows the FTIR spectra of the samples synthesized using different concentrations of ammonium bicarbonate in the range of 400–4000 cm^−1^. As can be seen, similar absorption peaks with small shifts are observed in the absorption spectrum of the samples, which demonstrate the same chemical structures of synthesized samples at different concentrations of ammonium bicarbonate. The peak at 1623 cm^−1^ confirms the presence of water in the sample and is based on the H–O–H bending vibration of H_2_O molecules. The wide peak in the region of 3200–3700 cm^−1^ is related to the stretching vibration of OH-, which is bonded to Al^3+^ ions^34,35^.

Also, the presence of broad absorption peaks in the range of 500–1000 cm^−1^ refers to the asymmetric stretching vibrations of the Al–O–Al bond^36,37^.

As mentioned, the positions of the FTIR absorption peaks obtained for a synthesized sample, are a fingerprint being used to determine the chemical bonds and phase of the products. γ-phase alumina has two well-known FTIR absorption peaks almost at 840, 558 cm^−1^ which can be a measure of the alumina formation in the γ-phase^38^. Therefore, the FTIR spectra of the alumina samples in the fingerprint range of 400 to 1350 cm^−1^ were deconvoluted to the Gaussian components by using Origin pro software to find the precise position of the peaks for the samples. The first FTIR absorption peaks of RA-0.6, RA-0.8, RA-1, and RA-1.2 are located at 571, 570, 573, and 576 cm^−1^, respectively. Also, the second ones are located at 820, 815, 837, and 813 cm^−1^, respectively. Hence, the characteristic FTIR absorption peaks of γ-phase alumina can be observed for all the synthesized samples, it can be concluded that the alumina formed in γ-phase. Therefore, γ-phase Al_2_O_3_ is created in all samples synthesized with different concentrations of ammonium and we will discuss the formation of nano alumina in the next subsection.

### The formation mechanism of nano-alumina

The formation of Al_2_O_3_ NPs occurs through the following chemical reactions^24^, which indicates the formation of NH_4_OH in the early stages of the synthesis process through the dissolution of ammonium bicarbonate in water (Eq. 1). Therefore, the concentration of NH_4_HCO_3_ has a direct relation to NH_4_OH production:

1 $${\text{NH}}_{{4}} {\text{HCO}}_{{3}} + {\text{H}}_{{2}} {\text{O}} \to {\text{NH}}_{{4}} {\text{OH}} + {\text{H}}_{{2}} {\text{O}} + {\text{CO}}_{{2}}$$

2 $${\text{Al}}\left( {{\text{NO}}_{{3}} } \right)_{{3}} + {\text{3NH}}_{{4}} {\text{OH}} \to {\text{Al}}\left( {{\text{OH}}} \right)_{{3}} \downarrow + {\text{3NH}}_{{4}} {\text{NO}}_{{3}}$$

3 $${\text{Al}}\left( {{\text{OH}}} \right)_{{3}} \to {\text{AlOOH}} \downarrow + {\text{H}}_{{2}} {\text{O}}$$

4 $${\text{AlOOH}} \to {\text{Al}}_{{2}} {\text{O}}_{{3}} + {\text{H}}_{{2}} {\text{O}}$$

According to references 16,39, the formation of Al(OH)_3_ is essential for creating alumina, which is provided during the interaction of NH_4_OH with aluminium nitrate (Eq. 2). In addition, in the present work, the addition of sodium hydroxide leads to the production of more Al(OH)_3_. When NaOH is added to the solution to adjust the pH, it is separated into Na and OH ions. It has been reported that both NaOH and ammonium bicarbonate act as precipitating agents in the synthesis process^24,40^. Both of them contribute to the production of OH^-^ ions in the solution that leads to the nucleation and later growth process. In this study, it was observed that the addition of NaOH to the precursor solution transforms the transparent solution into a milky white slurry due to the existence of solid particles in the solution. A similar observation has been reported in the synthesis of ZnO and alumina nanoparticles using NaOH^40,41^. Also, according to the molar ratios of the materials used in the synthesis process and corresponding chemical reactions, it can be suggested that both ammonium bicarbonate and NaOH contribute to precipitation and hence to the nucleation and growth of nanoparticles. However, since the synthesis efficiency increases with rising the amount of ammonium bicarbonate, the precipitating agent may be dominant here. Nevertheless, a more exact explanation demands further experiments to investigate the relationship between ammonium bicarbonate and NaOH as precipitating agents in the alumina synthesis process.

### XRD analysis

**Figure 3:**
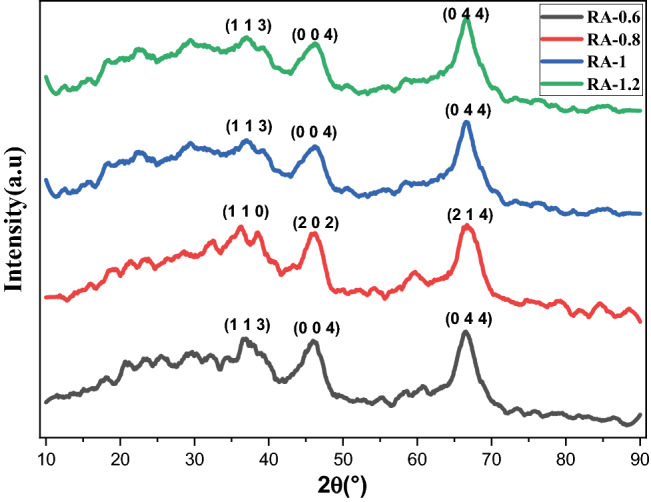
XRD patterns of alumina samples synthesized with different concentrations of ammonium bicarbonate including 0.6, 0.8, 1, and 1.2 g in 60 mL of deionized water.

The crystalline structures of the synthesized alumina samples were investigated by X-ray diffraction (XRD) analysis and the results in the range of 2θ = 10 to 90 degrees and with step sizes of 0.065° are depicted in Fig. 3. According to the results, some partly broad and low-intensity peaks can be observed in the XRD patterns of all the alumina samples, which demonstrates the small size of the crystal. In addition, identification of the crystal phase of the synthesized alumina samples by X’Pert high score software revealed that all samples are Al_2_O_3_ and formed in the Cubic phase. Moreover, the formation of the Gamma phase of alumina is verified through the comparison with the XRD patterns of γ-phase Al_2_O_3_ in other reported articles^6,21,22,37,38^. Therefore, XRD results confirm the FTIR results in the previous section.

The XRD patterns of the samples are refined by using MAUD software as a reliable The XRD data were used without any smoothing or filtering in the MAUD software and lattice constants were calculated, and the results are presented in Table 2. According to the results, a decreasing trend is observed in lattice constants with increasing the concentration of ammonium bicarbonate.

**Table 2:** Crystalline properties of Al_2_O_3_ nanoparticles synthesized with different concentrations of ammonium bicarbonate including 0.6, 0.8, 1, and 1.2 g.

Sample ID	Card number	Phase	Space group	Calculated lattice constants (Å)	Scherrer crystallite size (nm)
RA-0.6	96-201-5531	Cubic	F d -3 m	7.92	3.51
RA-0.8	01-073-2294	Cubic	F d -3 m	7.89	4.16
RA-1	96-120-0016	Cubic	F d -3 m	7.88	4.56
RA-1.2	96-120-0016	Cubic	F d -3 m	7.86	5.07

Also, the mean crystallite sizes of the alumina samples are calculated using Scherrer equation according to reference^42^ and presented in Table 2. The results reveal that the crystallite size increases with the increasing concentration of the precipitating agent.

### FESEM analysis

**Figure 4:**
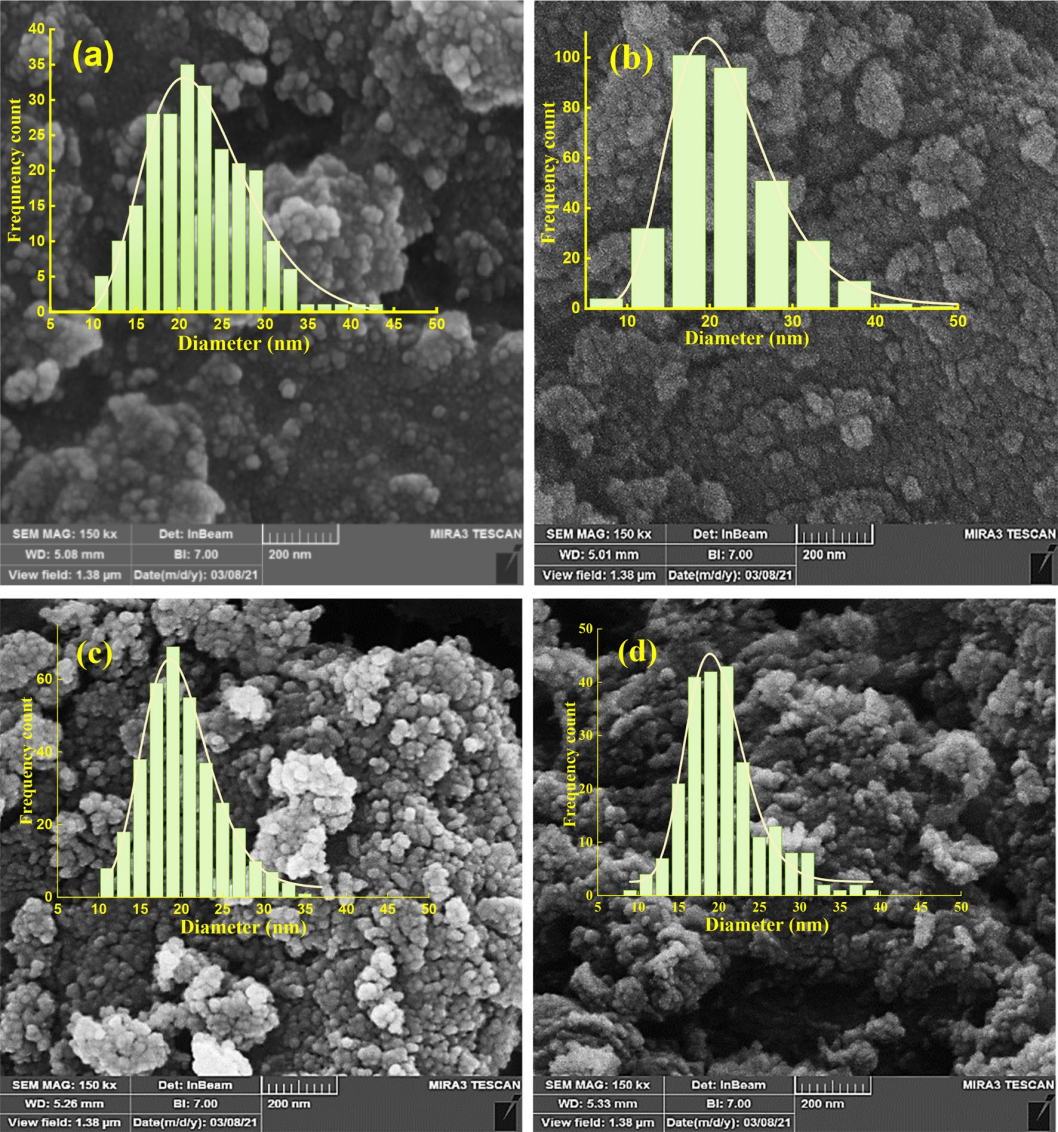
FESEM images and particle size histograms of alumina samples synthesized with different concentrations of ammonium bicarbonate labeled as (**a**) RA- 0.6, (**b**) RA- 0.8, (**c**) RA-1, and (**d**) RA-1.2.

Figure 4 displays the FESEM images of alumina samples synthesized at different concentrations of ammonium bicarbonate. It can be observed that the morphology of Al_2_O_3_ is quite independent of the concentration of ammonium bicarbonate, and agglomerated spherical nanoparticles are formed in all alumina samples. Also, it seems that using a higher concentration of the precipitating agent leads to producing more uniform nanoparticles. For more quantitative analysis, the average diameters and data dispersion of the alumina nanoparticles can be calculated using Digimizer software and fitting a log-normal fitting function in Origin pro software. The distribution histograms of nanoparticle diameters are depicted in the inset of the FESEM images for every synthesized alumina sample and the average diameters of nanoparticles are calculated and collected in Table 3. It is seen that the particle size follows only a slightly decreasing trend with increasing the concentration of ammonium bicarbonate and they are all below 24 nm in diameter. Furthermore, a significant difference can be observed between the crystallite size and particle size of the synthesized alumina NPs which can be concluded that the nanoparticles are polycrystalline that consist of small crystallites.

**Table 3:** The average diameter size and standard deviation of Al_2_O_3_ nanoparticles synthesized at different concentrations of ammonium bicarbonate.

Sample	D (nm)	Standard deviation (nm)	Percentage of standard deviation (%)
RA-0.6	23.10	0.93	4.06
RA-0.8	21.22	0.95	4.29
RA-1	19.54	0.45	2.26
RA-1.2	19.45	0.33	1.70

Furthermore, increasing the concentration of ammonium bicarbonate leads to more uniform nanoparticles, and the least data dispersion is obtained at the highest investigated concentration of the precipitating agent in sample RA-1.2.

**Figure 5:**
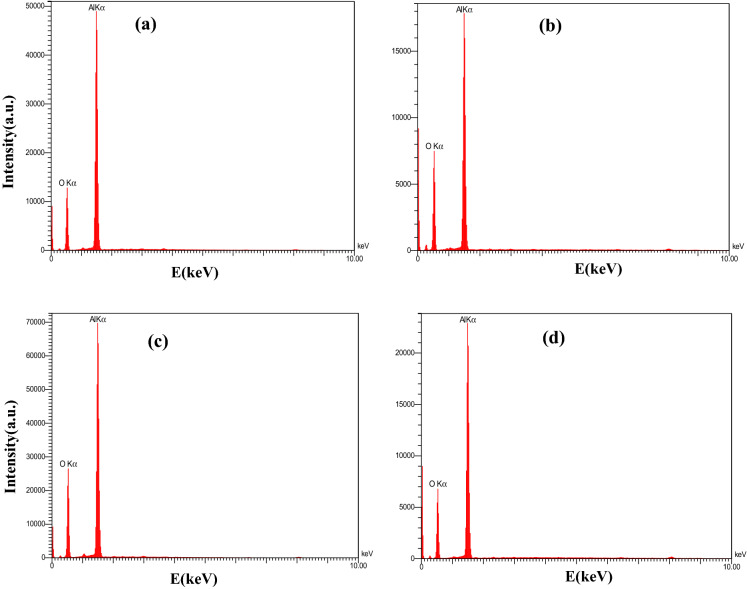
EDX spectra of Al_2_O_3_ nanoparticles synthesized using different concentrations of ammonium bicarbonate labeled as (**a**) RA- 0.6, (**b**) RA- 0.8, (**c**) RA-1, and (**d**) RA-1.2.

Also, elemental analysis of the samples was done using EDX technique by the FESEM machine and the results are presented in Fig. 5. EDX spectra of all nano-alumina samples synthesized at different concentrations of ammonium bicarbonate have two strong signals at 1.5 keV and 0.5 keV, which confirm the existence of Aluminium (Al) and Oxygen (O), respectively.

Furthermore, no extra peaks are observed in the spectra, which indicates the purity of all synthesized nano-alumina. For more detailed information, the weight percentage and atomic percentage of the elements in the nano-alumina samples are collected in Table 4.

**Table 4:** The weight percentage and atomic percentage of the elements (Al and O) in the Al_2_O_3_ nanoparticles synthesized at different concentrations of ammonium bicarbonate.

Sample	Elements	W%	A%
RA-0.6 g	Al	66.84	54.45
	O	33.16	45.55
RA-0.8 g	Al	58.8	45.84
	O	41.2	54.16
RA-1 g	Al	61.05	48.17
	O	38.95	51.83
RA-1.2 g	Al	65.22	52.65
	O	34.78	47.35

### DRS analysis

**Figure 6:**
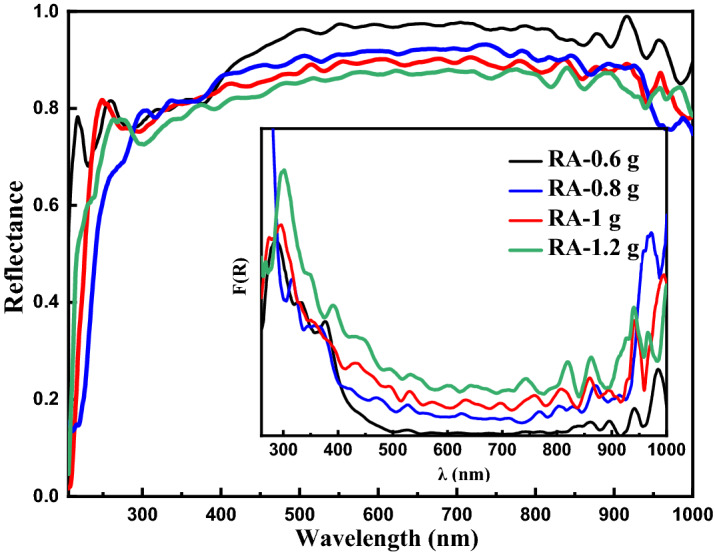
The diffuse reflectance spectra of alumina samples synthesized at different concentrations of ammonium bicarbonate including 0.6, 0.8, 1, and 1.2 g in 60 mL of deionized water. Calculated Kubelka–Munk of the alumina NPs is depicted in the inset of the figure.

Diffuse reflectance spectroscopy was done to achieve some information about the optical properties of the alumina NPs. As you can observe in Fig. 6, the concentration of ammonium bicarbonate significantly affects the reflectance of the synthesized alumina nanoparticles, so that the sample RA-0.6 has the highest reflectance in the visible and NIR regions and can be used as a high reflectance (HR) material in the regions. The Kubelka–Munk function, F(R), which is proportional to the absorption coefficient of the alumina and can be calculated from (F(R) = (1 − R)^2^/(2R)) is depicted in the inset of Fig. 6, where R in the function is the reflectance of alumina NPs. The results show that the absorption of the synthesized nano-alumina in the range of 500–850 nm experiences an increase with increasing the concentration of ammonium bicarbonate. The lowest reflectance/absorbance of all samples is located in the UV region. However, the absorption peak experiences a red-shift with increasing concentration of ammonium bicarbonate, and the highest absorption peak is observed for the sample RA-1.2.

**Figure 7:**
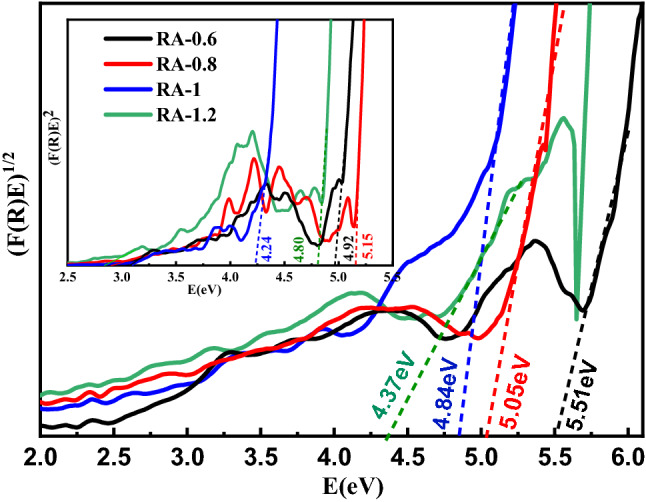
The Tuac’s plot and calculated indirect bandgaps of γ-Al_2_O_3_ nanoparticles synthesized at different concentrations of ammonium bicarbonate including 0.6, 0.8, 1, and 1.2 g in 60 mL of deionized water. Tuac’s plot and calculated direct bandgaps are shown in the inset of the figure.

To determine the direct and indirect bandgap values of the alumina nanoparticles, Tauc method (Eq. 5) can be used^43,44^.

5 $$\left( {F(R)E} \right)^{m} = B(E - E_{g} )$$

where E, B, and E_g_ are photon energy, a constant, and the material bandgap, respectively. m is 2 or 1/2 for the calculation of the direct or indirect bandgap energy, respectively and the direct and indirect bandgap energies can be determined from the intercept of (F(R)E)^2^ and (F(R)E)^1/2^ versus photon energy plots as shown in Fig. 7. The results declare that the concentration of ammonium bicarbonate significantly influences the direct and indirect bandgap energy of the synthesized nano-alumina. The calculated direct and indirect bandgap energies are listed in Table 5. All synthesized alumina NPs have bandgap energy of more than 4.24 eV (between 4.42 and 5.05 eV). Also, the lowest bandgap energy is obtained in the RA-1 sample. The bandgap energies of our synthesized samples are in the range of other reported studies. For example, Amirsalari et al. obtained the direct bandgap energy of 5.25 eV for alumina nanoparticles synthesized using a wet chemical method at pH = 8 and calcination temperature of 550 °C^45^. Koopi et al. also reported an energy bandgap of 5.46 eV for nano-alumina samples synthesized using a green method^46^.

**Table 5:** Direct and indirect energy band gap of Al_2_O_3_ nanoparticles synthesized at different concentrations of ammonium bicarbonate.

Sample ID	RA-0.6	RA-0.8	RA-1	RA-1.2
Direct bandgap (eV)	4.92	5.15	4.24	4.8
Indirect bandgap (eV)	5.51	5.05	4.84	4.37

Generally, structural parameters such as crystallite size and lattice parameters can be affected by the change in precursor concentration^47,48^. Also, bending energy depends on the lattice parameters of the samples and therefore can be modified by changing the concentration of ammonium bicarbonate in the synthesis process of alumina NPs. Also, the change of band gap can be related to the quantum confinement effect due to the increasing/decreasing trend of crystallites/nanoparticles sizes formed in the samples by increasing the concentration of ammonium bicarbonate.

### Specific surface area

The specific surface area (SSA) of the alumina samples can be estimated using XRD data in dense, monodisperse, and spherical particles approximation as follows^49^:

6 $$SSA = \frac{6000}{{\rho \times d}}$$

where ρ is the particle density of the alumina samples, which is determined using X’Pert high score software and d is the mean crystallite size calculated from XRD data. SSA values of the samples were calculated using Eq. (6) and the results are gathered in Table 6. Also, the SSA values of the alumina nanoparticles were measured by the multipoint BET (Brunauer–Emmet–Teller) method from N_2_ adsorption–desorption data and the results are presented in Table 6.

**Table 6:** Density calculated SSA, and BET SSA of alumina nanoparticles synthesized at different concentrations of ammonium bicarbonate including 0.6, 0.8, 1, and 1.2 g in 60 mL of deionized water.

Sample ID	Density	BET SSA (m^2^/g)	SSA (m^2^/g)
RA-0.6	3.59	317	474
RA-0.8	3.98	168	287
RA-1	3.64	264	394
RA-1.2	3.66	225	311

As we expected, the specific surface area of the alumina samples significantly depends on the concentration of ammonium bicarbonate used in the synthesis process.

Besides, the BET SSA and calculated SSA using XRD data has the same trend with increasing the concentration of ammonium bicarbonate. However, the estimated SSA values of the samples with these two methods are different, which may be due to the invalidity of the applied approximation. Indeed, the value of SSA changes with increasing the concentration of ammonium bicarbonate, and setting the pH value to 8 can be due to two competitive phenomena. First, increasing the ammonium bicarbonate leads to an increase in the SSA value. Second, setting the pH value while increasing the ammonium bicarbonate decreases the NaOH concentration in the solution and subsequently decreases the concentration of hydroxyl groups in the liquid. Also, according to reference 32, the SSA value of alumina nanoparticles directly relates to the concentration of hydroxyl groups in liquid and therefore falls with decreasing the NaOH concentration.

### Copper adsorption experiment

**Figure 8:**
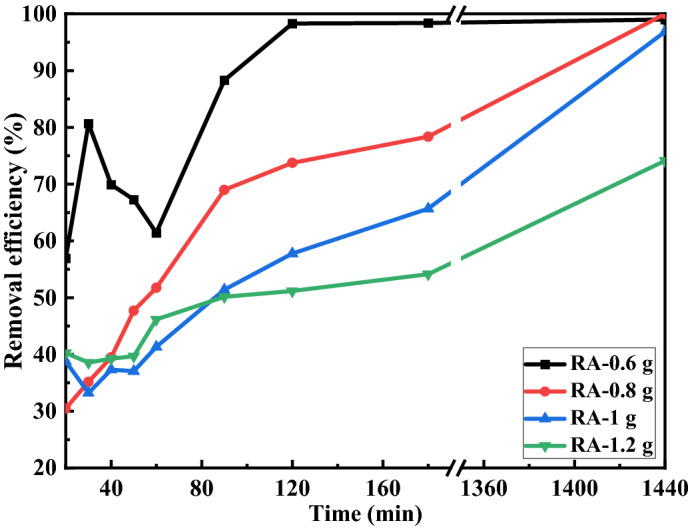
The percentage of Cu ions removal from water using synthesized nano-alumina at different concentrations of ammonium bicarbonate including 0.6, 0.8, 1, and 1.2 g in 60 mL of deionized water under the acidic condition (pH = 5.8).

Most nanoparticles have no desirable operation in acidic conditions. First, we investigate the Cu ions removal from the simulated water to investigate the effect of ammonium bicarbonate concentration on the water treatment in acidic conditions (pH = 5.8), and the results are presented in Fig. 8. The following equation was used to calculate the efficiency of Cu ions removal.

7 $${\text{Removal}}\;{\text{efficiency}}\;\% = \left( {C - C_{0} } \right) \times 100/C_{0}$$

where, C_0_ and C are the initial concentration of copper in solution and its concentration at time t, respectively.

According to Fig. 7, the concentration of ammonium bicarbonate significantly affects the ability of Cu ions removal by the synthesized alumina nanoparticles, and the highest efficiency is obtained using the sample RA-0.6, and copper ions removal% reaches 98.2% in 120 min. Also, copper ions removal% at the same time is 73.5%, 57.8%, and 51.2% for RA-0.8, RA-1, and RA-1.2, respectively, which is an acceptable Cu^2+^ removal from water.

The efficiency of Cu ions removal of RA-0.6 from water is interesting to compare with the reported results for Cu removal% by alumina nanoparticles in the literature. For example, Ren. yu Wang et al. have synthesized alumina nanostructures by sol–gel method and used it to absorb copper metal contaminants from water^38^. The maximum adsorption capacity of alumina nanoparticles for copper metal was 88.7 mg/L (about 65%), which indicates the greater capacity of sample RA-0.6 for Cu^2+^ removal from water.

The difference in Cu removal% of the synthesized nano-alumina samples can be related to the high SSA of the RA-0.6. High specific surface area (SSA) leads the high active sites, which require adsorption, which is one of the most significant adsorbent characteristics. The direct relation between the performance of material adsorption and surface area was previously reported in Reference^50^. However, the electrostatic force which affects the results should be considered too.

**Figure 9:**
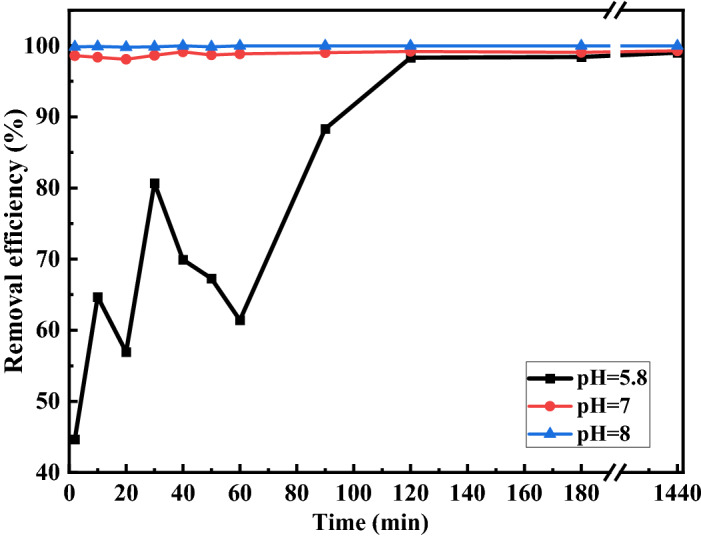
The percentage of Cu ions removal from water using RA-0.6 sample in acidic, neutral, and alkaline conditions (pH = 5.8, 7, and 8).

As mentioned, the RA-0.6 sample has the highest Cu ions adsorption at the investigated acidic condition. So, the Cu ions removal of the RA-0.6 sample was studied in neutral, acidic, and alkaline conditions (pH = 5.8, 7, and 8), and the results are depicted in Fig. 9. According to the results, the efficiency of the sample in neutral and alkaline conditions are significantly higher than the acidic condition and most Cu ions are removed from water in less than 10 min in neutral and alkaline conditions while it takes 120 min to reach the efficiency of 98.2% in acidic condition.

Previous works on alumina nanoparticles have been reported and shown a better Cu ions removal of the nanoparticles in alkaline conditions^51^. However, our efficiency in acidic, neutral, and alkaline conditions is significantly admirable and the alumina nanoparticles can be an excellent candidate for water treatment and Cu ions removal from water in every condition.

The kinetics of Cu adsorption on the Al_2_O_3_ nano-particles are explored using pseudo-first-order and pseudo-second-order kinetics to further understand the adsorption mechanism. The pseudo-first-order and pseudo-second-order kinetics are based on the adsorption capacity of the adsorbent. The pseudo-first-order reaction can be calculated by the following equation:

8 $$- Ln(C_{t} /C_{0} ) = k_{app} t$$

where *C*_*0*_ is the initial concentration and *C*_*t*_ is the time-dependent residual concentration of Cu ion solution and *k*_*app*_ is the rate of Cu removal from water^52,53^.

The pseudo-second-order reaction model is presented as follows:

9 $$t/Q_{t} = 1/k_{2} Q_{e}^{2} + t/Q_{e}$$

where *k*_2_ (g mg^−1^ min^−1^) is a rate constant of the pseudo-second-order kinetic model, and *Q*_*e*_ and *Q*_*t*_ (mg g^−1^) are the amounts of Cu adsorbed per unit mass of the nano alumina samples at the equilibrium and time t, respectively. *Q*_*e*_ can be calculated using the following equation^18^:

10 $$Q_{e} = (C_{0} - C_{e} )V/W$$

where *V* (L) is the volume of the Cu solution, *W* (g) is the mass of inserted adsorbent in the experiment, and *C*_*e*_ is the concentration at the equilibrium.

**Figure 10:**
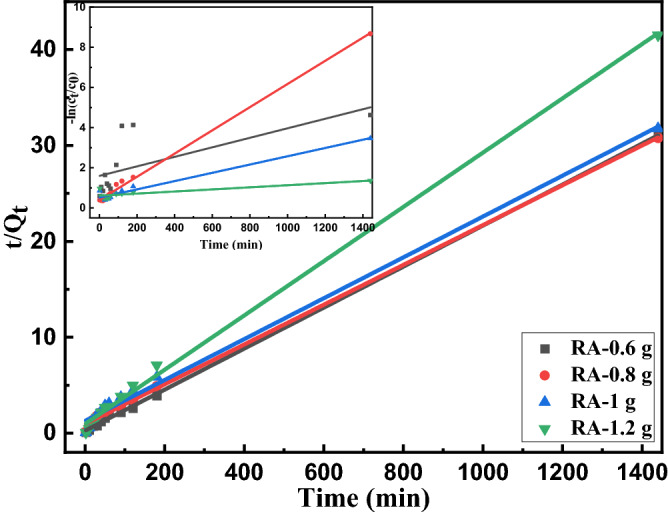
Adsorption kinetics of Cu ion on the Al_2_O_3_ nano-particles for the samples using pseudo-second-order kinetics (pH 5.8, initial Cu concentration of 46.79 mg L-1) with Pseudo-first-order in the inset.

The plots of $t/Q_{t}$ versus contact time are depicted in Fig. 10 for the synthesized nano alumina at pH = 5.8 and straight lines are fitted to find out the kinetics of Cu adsorption on the synthesized nano alumina in the range of 2 to 1440 min. Also, the plots of − ln (*C*_0_*/C*_*t*_) versus contact time are represented in the inset of Fig. 10 for the samples, and straight lines are fitted to the data. Rate constants of the pseudo-first and -second-order kinetic model (*k*_*app*_ and *k*_2_) of the samples for Cu removal from acidic water are calculated and gathered in Table 7. As it can be observed, the correlation coefficients of the pseudo-first and second-order reaction, R^2^, of sample RA-0.6 are 0.436 and 0.999, respectively which means the kinetics of Cu ion adsorption by the Al_2_O_3_ nanoparticles follows the pseudo-second-order reaction or chemisorption. However, these correlation coefficients for other alumina NPs have partly the same correlation coefficients for the pseudo-first and second-order models which means both physisorption and chemisorption involve in Cu ion removal from water by other samples. Previously, Ren. yu Wang et al. reported the pseudo-second-order kinetics model for Cu adsorption by their synthesized alumina nanoparticles^49^ and the results are consistent with this study in the case of the adsorption mechanism. It is also worth mentioning that the largest value of *k*_*2*_ in the acidic condition is obtained for the sample RA-06 and this Al_2_O_3_ sample can be a good candidate for Cu ions removal from alkaline, neutral, and even acidic water.

**Table 7:** Parameters of the adsorption isotherm model for copper adsorption by our synthesized nano-alumina in acidic simulated water (pH = 5.8).

Sample	Pseudo-first-order		Pseudo-second-order	
	*k *_*app*_ (min^−1^)	*R*^2^	*k*_2_ (g mg^−1^ min^−1^)	*R*^2^
RA-0.6	0.00237	0.436	16.9	0.999
RA-0.8	0.00577	0.996	4.51	0.998
RA-1	0.00206	0.968	3.50	0.994
RA-1.2	5.21E-04	0.699	8.32	0.997

**Figure 11:**
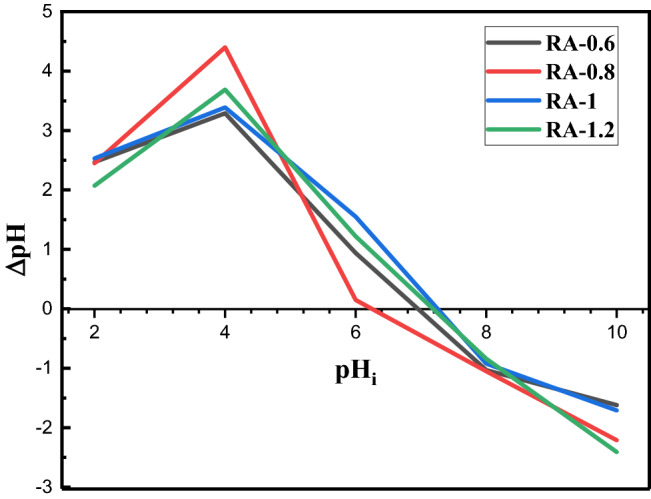
The point of zero charge (PZC) for alumina nanoparticles synthesized with different concentrations of ammonium bicarbonate.

The point of zero charge (PZC) should be estimated to describe the effects of pH value on the water treatment or Cu ion removal which deals with colloidal flocculation or the effect of the net charge of the alumina samples on the phenomena. Therefore, the difference between the final (after 48 h) and initial pH values of the solutions containing synthesized alumina samples, ΔpH, are plotted versus the initial pH value, pH_i_, in Fig. 11. The intersection of each curve to the horizontal axis gives the pH of PZC, PH_PZC_, which is different for different alumina samples synthesized at different concentrations of ammonium bicarbonate. PZC of RA-0.6, RA-0.8, RA-1, and RA-1.2 samples are 6.9, 6.2, 7.3, and 7.2, respectively. As you could observe, the lowest values are obtained for RA-0.8 and RA-0.6 samples, and also the PZC values rise with increasing the concentrations of ammonium bicarbonate from sample RA-0.8 to RA-1 and then no significant change can be observed in pH_PZC_ from sample RA-1 to RA-1.2. As it can be realized from Fig. 8, the highest Cu removal% is obtained for RA-0.6 (with the highest BET SSA 317 m^2^/g) followed by RA-0.8 (with the lowest pH_PZC_ = 6.2). The results can be interpreted by the SSA and PZC values of the alumina samples. High specific surface area (SSA) leads to the highly required active sites, which is one of the most significant adsorbent characteristics. The direct relation between the performance of material adsorption and surface area was previously reported in reference^50^. It can explain the highest Cu ion removal of RA-0.6 obtained in pH = 5.8 but does not explain the results of water removal for RA-0.8 with the lowest SSA (BET SSA = 168 m^2^/g) in the samples.

The net surface charge of the synthesized alumina is positive/negative before/after pH of PZC. Also, cations such as copper are strongly adsorbed to the surface of alumina NPs with negative surface charges because of attractive electrostatic forces as confirmed by Fig. 9 in neutral and alkaline conditions (pH = 7, and 8) for RA-0.6 with the pH_PZC_ 6.9. However, the repulsion force between Cu ions and positive surface charges of NPs (before pH_PZC_) can affect water removal which is a major problem in the treatment of acidic waters. The acidic water with a pH of 5.8 is near the pH_PZC_ of RA-0.8 which means no significant electrostatic forces affect the adsorption process of Cu^2+^ by the samples and it can be the reason for the high adsorption of the sample in the acidic pH. Finally, the small difference between the selected pH of acidic water and pH_PZC_ of the RA-0.6 and also the highest adsorption sites of the NPs due to the highest SSA of the sample leads to the highest Cu^2+^ removal from the acidic water. As we expected, the attractive electrostatic force between Cu^2+^ ions and the negative surface charge of RA-0.6 at pH = 8 leads to Cu removal% of 100% in the first seconds of the contact in Fig. 9. Therefore, the RA-0.6 samples can be proposed as an excellent candidate for the treatment of water with a pH of 5.8 or more.

## Conclusion

In summary, the alumina nanoparticles were prepared by the co-precipitation method with high specific surface areas (SSA) and were employed as adsorbents for Cu adsorption. In this work, ammonium bicarbonate was used as a precipitating agent in different masses, and the effects of ammonium bicarbonate concentration on the structural and optical properties of the synthesized nano-alumina were investigated. In all concentrations of the precipitating agent, Al_2_O_3_ nanoparticles were successfully produced. Also, the concentration of the precipitation agent affects the nanoparticles and crystallite sizes. Furthermore, the highest specific surface area (317 m^2^/g) (SSA) as well as the highest percentage of pollutant adsorption in acidic simulated water were obtained with nano-alumina synthesized with the precipitating agent mass of 0.6 g. Indeed, the adsorption of copper ions and removal efficiency depend on the contact time and pH of the solutions. It rises with the increase of contact time and the best Cu removal is obtained in neutral and alkaline conditions. However, the removal efficiency of the nano-alumina samples synthesized by the precipitating agent mass of 0.6 g is significantly high in the acidic medium and reaches 98.7% in a contact time of 120 min, which is a high value in an acidic condition and suggests the nano-alumina as an excellent candidate even for acidic water treatment. Also, the study of the adsorption mechanism declares that all synthesized nano-alumina follows the pseudo-second-order kinetics model.

## Data Availability

All data included in this paper are available upon request by contact with the corresponding author.
